# Natural product, bilobalide, improves joint health in rabbits with osteoarthritis by anti-matrix degradation and antioxidant activities

**DOI:** 10.3389/fvets.2022.1034623

**Published:** 2022-10-20

**Authors:** Tianwen Ma, Hong Chen, Hongri Ruan, Liangyu Lv, Yue Yu, Lina Jia, Jinghua Zhao, Xin Li, Yuxin Zang, Xinyu Xu, Jiantao Zhang, Li Gao

**Affiliations:** ^1^College of Veterinary Medicine, Northeast Agricultural University, Harbin, China; ^2^College of Animal Science and Technology, College of Veterinary Medicine, Zhejiang Agriculture and Forestry University, Hangzhou, China; ^3^Heilongjiang Key Laboratory of Animals Disease Pathogenesis and Comparative Medicine, Harbin, China

**Keywords:** bilobalide, Nrf2/HO-1 pathway, oxidative stress, osteoarthritis, papain

## Abstract

Osteoarthritis (OA) is a common chronic musculoskeletal disease reported in veterinary clinics that severely reduces the quality of life of animals. The natural product, bilobalide, has positive effects on chondroprotection but its exact mechanism of action is unclear. This study aimed to investigate the antioxidant and anti-matrix degradation activities of bilobalide in a rabbit model of OA and its protective effects on joints. We also investigated the possible mechanisms underlying these effects. The rabbit OA model was established by intra-articular injection of 4% papain. Thirty healthy male New Zealand rabbits were randomly divided into control, untreated OA, Cel (100 mg/kg celecoxib intervention as a positive control), BB-L and BB-H (40 mg /kg and 80 mg /kg bilobalide gavage treatment, respectively) groups. Two weeks after surgical induction, bilobalide or celecoxib was administered by gavage daily for 8 weeks. After 8 weeks of bilobalide intervention, cartilage macroscopic observation and histopathological images showed alleviation of cartilage damage after bilobalide treatment, and the Osteoarthritis Research Society International (OARSI) score was significantly lower than that in the OA group. Bilobalide reduced the expression of metalloproteinase 3 (MMP-3) and MMP-13 in cartilage tissue of OA rabbits and reversed the levels of serum C-telopeptides of type II collagen (CTX-II), cartilage oligomeric matrix protein (COMP), interleukin 1(IL-1), and tumor necrosis factor (TNF-α). Bilobalide (80 mg/kg) could improve the biomechanical properties and microstructural changes in subchondral bone in the early stage of OA in rabbits, thereby delaying subchondral bone damage. Mechanistically, bilobalide exerted antioxidant and anti-matrix degradation effects by upregulating the oxidative stress signaling Nrf2/HO-1 pathway and inhibiting cartilage degeneration in rabbit OA. We thus speculate that bilobalide supplements recovery from OA damage.

## Introduction

Knee osteoarthritis (OA) is common chronic musculoskeletal disease, which can cause pain, limited mobility, and even disability (1, 2). To date, no disease-modifying therapies for OA exist due to insufficient understanding of the pathogenesis of knee OA (3). Non-steroidal anti-inflammatory drugs (NSAIDs) that, temporarily relieve pain and have no preventive or curative impact on cartilage deterioration, are among the currently available treatments for OA (4). Due to these restrictions, new, side-effect-free strategies are needed to strengthen the cartilage and stop its degeneration while enhancing overall joint health.

Bilobalide, a sesquiterpenoid derived from *Ginkgo biloba* L. extract (5), exerts anti-inflammatory (6), anti-apoptotic (7), antioxidant (8), neuroprotective (9), and microcirculation-improving properties (10). Recent research indicates that bilobalide can reduce anterior cruciate ligament transection (ACLT)-induced OA in rats through its action on chondrocytes *via* the AMPK/SIRT1/mTOR pathway to reduce inflammation (11). By inhibiting microRNA-125a, bilobalide reduces IL-17-induced inflammatory damage in the ATDC5 chondrocytes (12). Although these studies suggest that bilobalide has potential protective effects for OA, the specific mechanism remains unclear and experimental validation in animal models is lacking. Therefore, we established a rabbit OA model by injecting papain intra-articularly to observe the therapeutic impact of bilobalide, crucial for assessing its safety.

Joint inflammation and oxidative stress are directly linked to the development of OA (13–15). Upregulation of cellular antioxidant defense mechanisms in chondrocytes in several *in vitro* and *in vivo* investigations shows repression of catabolic genes expression and enhanced joint health (16–18). The master transcriptional regulator of the cellular antioxidant defense system is nuclear factor erythroid-2 related factor 2 (Nrf2). Under physiological conditions, cytoplasmic Nrf2 is sequestered by Keap1, following which it dissociates and translocates to the nucleus, where it binds to antioxidant response elements (AREs) and stimulates the transcription of HO-1 and other defense enzymes (19–21). Nrf2 activation has prevention or trerapeutic against OA. (22). Therefore, we hypothesized that the protective effect of bilobalide on papain-induced OA in rabbits was linked to the overexpression of Nrf2/HO-1.

## Materials and methods

### Experimental design

**Figure 1:**
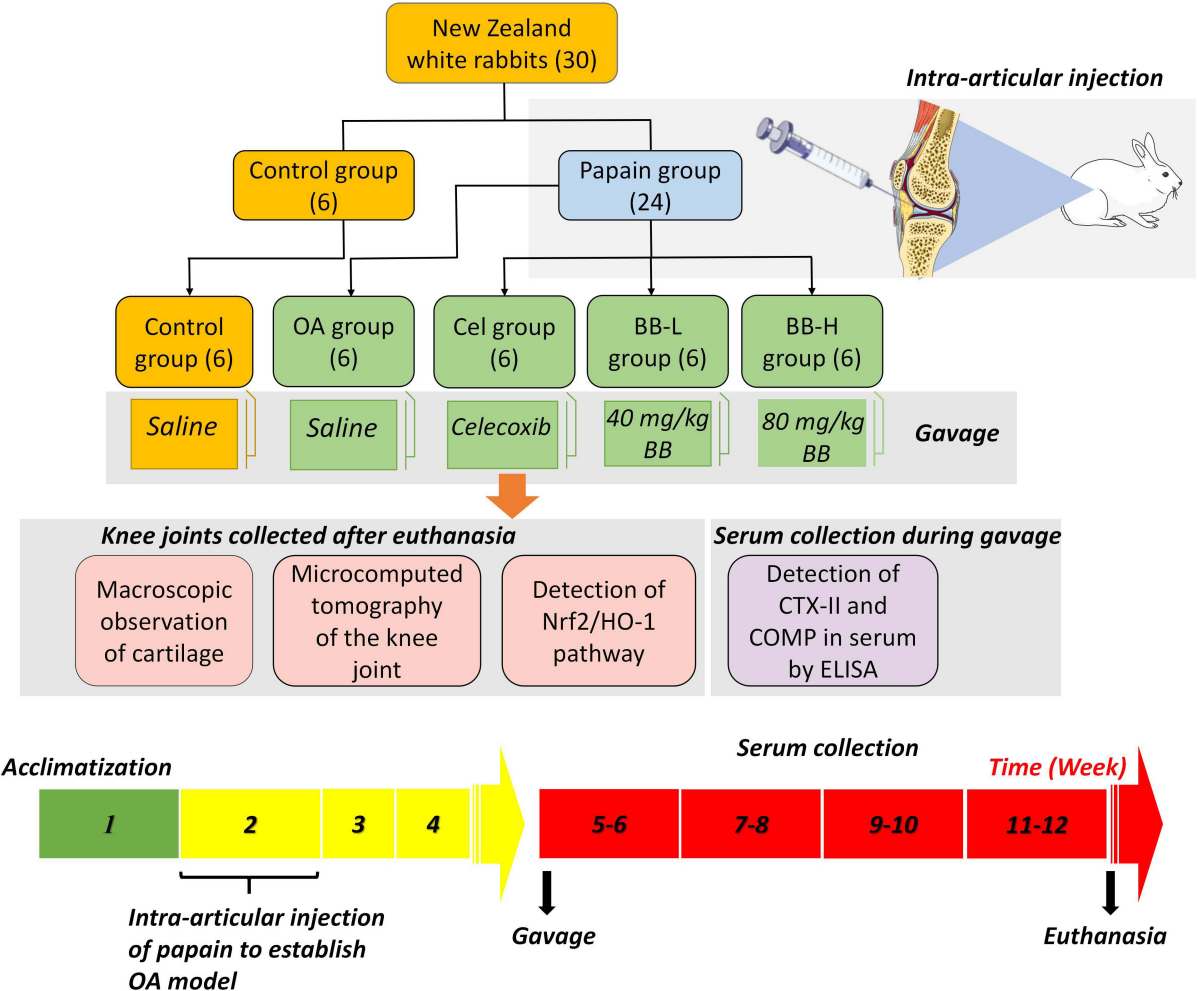
Papain-induced rabbit osteoarthritis model: experimental grouping, dosing frequency, and experimental design.

For the experiment, 30 adult male New Zealand white rabbits aged 3 months were chosen. The animals were acquired from the Animal Experiment Center of the Second Affiliated Hospital of the Harbin Medical University (Harbin, China), and housed in a controlled environment (light/dark, 12/12 h, and temperature 23 ± 1°C). Each rabbit was kept in a separate cage with free access to water, and normal chow and animals were inspected daily. The rabbits were allowed to acclimatize for a week before the experimental treatment. Rabbits were randomly divided into Control (0.9% NaCl treatment, *n* = 6) and papain (intra-articular injection of 4% papain to induce OA model, *n* = 24) groups. The latter was further categorized into 4 subgroups (6 animals in each group) as follows: OA (gavage with normal saline), Cel (positive control group, administered 100 mg/kg celecoxib which was purchased from Pfizer Pharmaceuticals [New York, USA]), BB-L (administered 40 mg/kg bilobalide to treat rabbit OA), and BB-H (80 mg/kg of bilobalide was administered to treat OA in rabbits) (Figure 1). The dose selection of bilobalide was based on the results of preliminary experiment. Recent standards for best practice in natural product pharmacological research requirements that are deemed relevant were considered (23, 24). The Laboratory Animal Welfare and Ethics Committee of Northeast Agricultural University (#NEAU-2022-01-0025-1) approved the procedure for animal surgery and treatment in this study. Every attempt was made to minimize the number of animals utilized and their suffering.

### Induction and treatment of OA

Chengdu Must Bio-technology Co., Ltd. (Chengdu, China) supplied the bilobalide (purity ≥ 98%). OA modeling was done based on previously described protocol (25). Briefly, the rabbits in the papain group received injections of 0.1 ml 4% papain (Solarbio, China) on the first, fourth, and seventh days in the cavity of the right knee. No manipulation was performed on the left hind knee of the animal. After each intra-articular injection, the animals were allowed to walk freely in the cage. In the Cel, BB-L and BB-H groups, 7 days after the last injection of papain, the animals were administered 100 mg/kg celecoxib, 40 mg/kg bilobalide, and 80 mg/kg of bilobalide, respectively once a day for 8 weeks (Figure 1). The gavage volume of celecoxib or bilobalide for each rabbit was 3 ml. In the control group, rabbits received 0.9% NaCl by gavage. After 8 weeks of drug intervention, animals were euthanized by 20 ml/kg air embolization (30 mg/kg 3% pentobarbital sodium *via* ear vein) under anesthesia. The blood samples were collected, centrifuged at 1,000 × g for 20 min, and the supernatant was collected for ELISA. Histological, western blotting, and qRT-PCR were performed on the knee cartilage.

### X-ray observation of the rabbit knee joint

X-rays scans of supine rabbits were captured using a direct radiography system (Longsafe, China, Animal Clinical Teaching Hospital of Tohoku Agricultural University). X-ray images were graded according to the Kellgren-Lawrence (KL) system (26).

### Microcomputed tomography (μCT)

On the Bruker Micro-CT Skyscan 1276 system (Kontich, Belgium), specimens were scanned. The scan settings were as follows: Voxel size, 15.067119 m; resolution, medium, and integration time, 403 ms. The calcium hydroxyapatite (CaHA) phantom provided by the manufacturer served as the standard for density measurements. The evaluation program from the manufacturer was used for the analysis. NRecon was used for the reconstruction (version 1.7.4.2). 3D images were obtained from contoured 2D images based on the distance transformation of the grayscale original images (CTvox; version 3.3.0). The CT Analyzer (version 1.18.8.0) software was used for 3D and 2D analysis. In the region of interest (ROI), analyses of the bone's microarchitecture were performed. Quantitative analysis of percent bone volume (BV/TV), mean trabecular number (Tb.N), mean trabecular thickness (Tb.Th) and mean trabecular separation (Tb. Sp) were conducted for each sample.

### Macroscopic observation

To evaluate the degree of articular cartilage destruction, images of the rabbit joint's femoral condyle and tibial plateau were captured (Nikon D7500 AF-S 18-140 mm f/3.5-5.6G ED VR camera). On a scale of 0–4, the degree of articular cartilage degeneration was rated (27). An observer who was blind to the treatment groups made macroscopic observations.

### Histological analysis

The tibias was dissected from each group and preserved in 10% buffered formalin for 72 h, followed by 3 weeks of decalcification in a 0.5 M EDTA solution. Subsequently, sagittal sections of 5 m thickness were obtained from each joint and immersed in paraffin. Hematoxylin-eosin (H&E) staining was performed. The degree of pathological damage to knee cartilage was assessed using the Osteoarthritis Research Society International (OARSI) score (28), with higher scores indicating higher severity of cartilage degeneration. Two seasoned observers who were blind to the treatment stained all of the sections in one batch and scored them.

### Immunohistochemistry

Antigen retrieval was performed on deparaffinized sections using the Target Retrieval Solution (Dako, USA). After quenching in Dako REAL™ Peroxidase Blocking Solution (Dako, USA), the slides were incubated with primary antibodies against Nrf2 (Affinity, China), HO-1 (Affinity, China), MMP-3 (Proteintech, China), and MMP-13 (Proteintech, China) overnight at 4°C. Following three PBS washes, the sections were incubated with the corresponding secondary antibody for 50 min followed by an examination on the liquid diaminobenzidine (DAB) + substrate-chromogen system. Leica photomicroscope (Leica, Germany) was used to capture the images.

### Western blot analysis

Western blot analysis was conducted as described previously (29). Rabbit knee joints' cartilage tissue samples from each group were dissected, homogenized, and the tissue homogenates were immediately centrifuged twice at 12,000 × g for 15 min at 4°C to isolate supernatant for subsequent western blot analysis. The total protein extracted was quantified using the bicinchoninic acid protein assay kit (BCA kit, Thermofisher, USA). Equal proportions of proteins were transferred to polyvinylidene fluoride (PVDF) membranes following separation by 10% SDS-PAGE (Beyotime,China). The membrane was blocked in 5% bovine serum albumin (BSA, Sigma, USA) followed by incubation with primary antibodies against Nrf2, HO-1, MMP-3, and GAPDH (Affinity, China) overnight at 4°C. The next day, the membranes were incubated with horseradish peroxidase (HRP)-conjugated secondary antibody (goat anti-rabbit/anti-mouse IgG, Affinity, China) for 1 h at room temperature. Western blotting analysis was performed by visualization of bands using the ECL chemiluminescence reagent (Vazyme, China). Using the Tannon automated gel image analysis technology (Shanghai, China), protein bands were detected. To examine the relative expressions of proteins, the Image J software was utilized.

### Quantitative real-time PCR (qRT-PCR)

Total RNA was extracted from the cartilage tissue of the knee joint using the TRIzol (Invitrogen, USA). RNA was reverse transcribed into cDNA using a cDNA synthesis kit (Vazyme, China) following the manufacturer's instructions. Gene expression was quantified using the ChamQ Universal SYBR qPCR Master Mix (Vazyme, China). The primer sequences used in this study are listed in Table 1. Expression was evaluated in triplicate for each gene to reduce the processing error. Using the 2^−ΔΔCt^ method, relative gene expressions were evaluated by normalizing the levels of target gene to that of glyceraldehyde-3-phosphate dehydrogenase (GAPDH).

**Table 1:** The primer sequences used in qRT-PCR assay.

**Gene**	**Forward primer (5^′^-3^′^)**	**Reverse primer (5^′^-3^′^)**
Nrf2	TTAGTGCTTTTGAGGATTCTTTCGG	AATTCTGTGCTTTCAGGGTGGTTCT
HO-1	CAGGTGACTGCCGAGGGTTTTA	GGAAGTAGAGCGGGGCGTAG
MMP-3	ATGGACCTTCTTCAGCAA	TCATTATGTCAGCCTCTC
MMP-13	AGGAGCATGGCGACTTCTAC	TAAAAACAGCTCCGCATCAA
GAPDH	ACTGGCGTCTTCACCACCAT	AAG GCCATGCCAGTGAGCTT

### Enzyme-linked immunosorbent assay (ELISA)

After 8 weeks of administration, blood samples were collected from rabbits in each group, and ELISA was performed to detect the levels of OA biomarkers, CTX-II and COMP, and inflammatory factors, IL-1 and TNF-α, in rabbit serum. Commercially available ELISA kits were purchased from Shanghai Enzyme-linked Biotechnology Co., Ltd. The ELISA kit reagents were equilibrated at room temperature for 30 min before the test, and the serum loading time was kept under control within 5 min. The manufacturer's instructions were properly followed. For each set of samples, three replicate wells were utilized, and the OD values were calculated. The standard curve regression equation was computed using the ELISA Calc program. To calculate the relevant concentration, the model was fitted to a four-parameter logistic curve. All analyses were performed by the Heilongjiang Key Laboratory of Animals Disease Pathogenesis and Comparative Medicine.

### Statistical analysis

Statistical analyses were performed using the SPSS software (version 19.0 for Windows, SPSS, Chicago, IL, USA). The results are summarized as mean ± standard deviation (SD). For two-group comparisons, data were analyzed by a two-tailed Student's *t-*test. For multiple group comparisons, ANOVA was performed followed by the least significant difference (LSD) test for significance. *p*-value < 0.05 was considered statistically significant.

## Results

### X-ray examination

**Figure 2:**
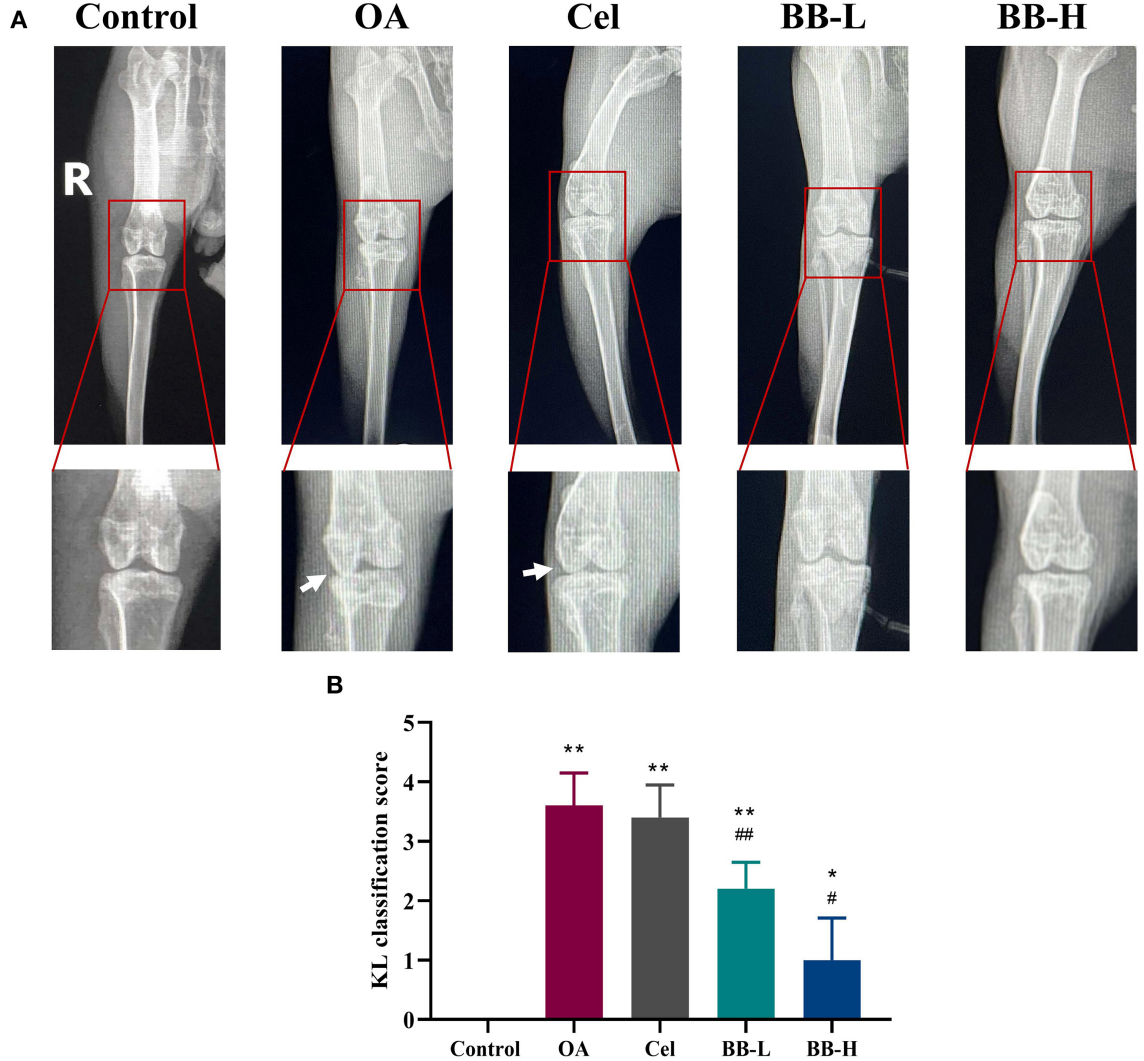
X-ray images of the knee joints of rabbits in each group after 8 weeks of bilobalide treatment. Arrows depict the knee joint's articular cartilage damage and shrinken joint space. **(A)** X-ray images of the knee in each group. **(B)** KL classification score for the knee in each group. All data are presented as mean ± SD (*n* = 3). * *p* < 0.05 and ** *p* < 0.01 vs. control group; ^#^ *p* < 0.05 and ^##^ *p* < 0.01 vs. OA group.

Figure 2A shows a representative X-ray image of the rabbit knee joint. The articular surfaces of the femoral condyle and tibial plateau display smooth and tidy dense shadows with smooth and clear outlines. There were no significant changes in the joint space in the control group. The shadow area of the joint soft tissue was larger in the OA group, the articular surface was uneven and rough, the joint space was smaller, and osteophytes were present. The osteophytes and edema in the knee joint vanished in the Cel group, but remained narrow with a rough surface. The femoral condyle and tibial plateau of the rabbits in the BB-L and BB-H groups treated with bilobalide showed smoother articular surfaces, and the joint space improved drastically as compared to the OA group. The KL scores of the OA, Cel, BB-L, and BB-H groups were considerably higher than those of the control group (control vs. OA, Cel, BB-L *p* < 0.0001; control vs. BB-H *p* = 0.0400), as shown in Figure 2B. Relative to the OA group, the KL classification scores of the BB-L and BB-H groups were considerably lower (OA vs. BB-L *p* = 0.0026; OA vs. BB-H *p* < 0.0001) (Figure 2B).

### Micro-CT examination

**Figure 3:**
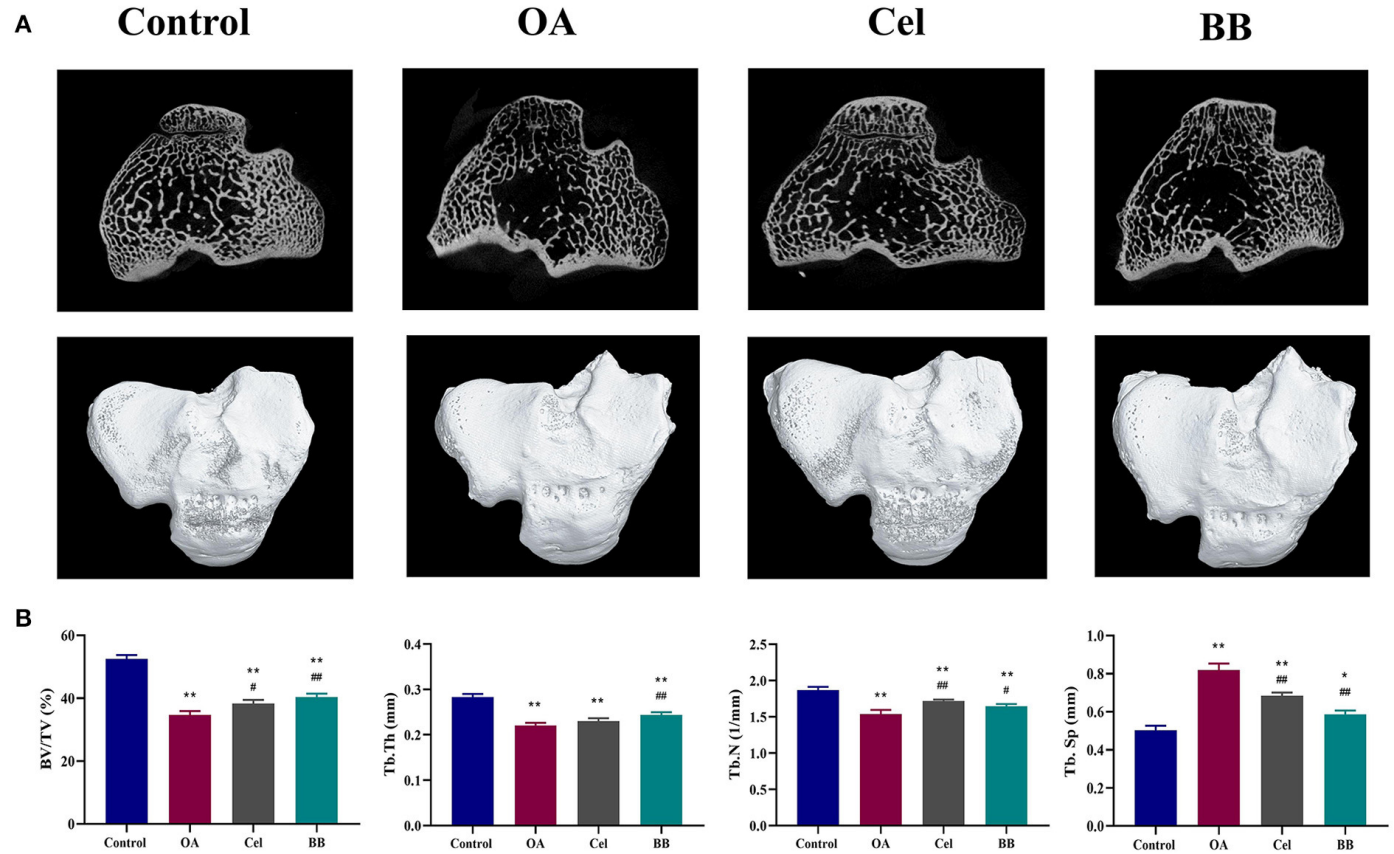
After 8 weeks of bilobalide treatment, a micro-CT study of the proximal tibia in different groups was performed. **(A)** Representative Micro-CT 3D images show bone microarchitecture of the proximal tibia in different groups. Scale bar = 5 mm. **(B)** Quantification of BV/TV, Tb.Th, Tb.N, and Tb.Sp in different groups. All data are presented as mean ± SD (*n* = 3). * *p* < 0.05 and ** *p* < 0.01 vs. control group; ^#^ *p* < 0.05 and ^##^ *p* < 0.01 vs. OA group.

A 3D rendering of the proximal tibia is shown in Figure 3A. As shown in Figure 3B, the OA, Cel, and BB (80 mg/kg of bilobalide was administered to treat OA in rabbits) groups showed substantial reductions in BV/TV (*p* < 0.0001), Tb.Th (control vs. OA, Cel *p* < 0.0001; control vs. BB *p* = 0.0003), and Tb.N relative to the control group (control vs. OA *p* < 0.0001; control vs. Cel *p* = 0.0081; control vs. BB *p* = 0.0006). The OA (*p* < 0.0001), Cel (*p* < 0.0001), and BB groups (*p* = 0.0121) exhibited greater Tb.Sp values than the control group. The BV/TV (*p* = 0.0018), Tb.Th (*p* = 0.0090), and Tb.N (*p* = 0.0483) values in the BB group were substantially higher than those in the OA group, although the Tb.Sp value of the BB group was lower than that of the OA group (*p* < 0.0001).

### Macro observations

**Figure 4:**
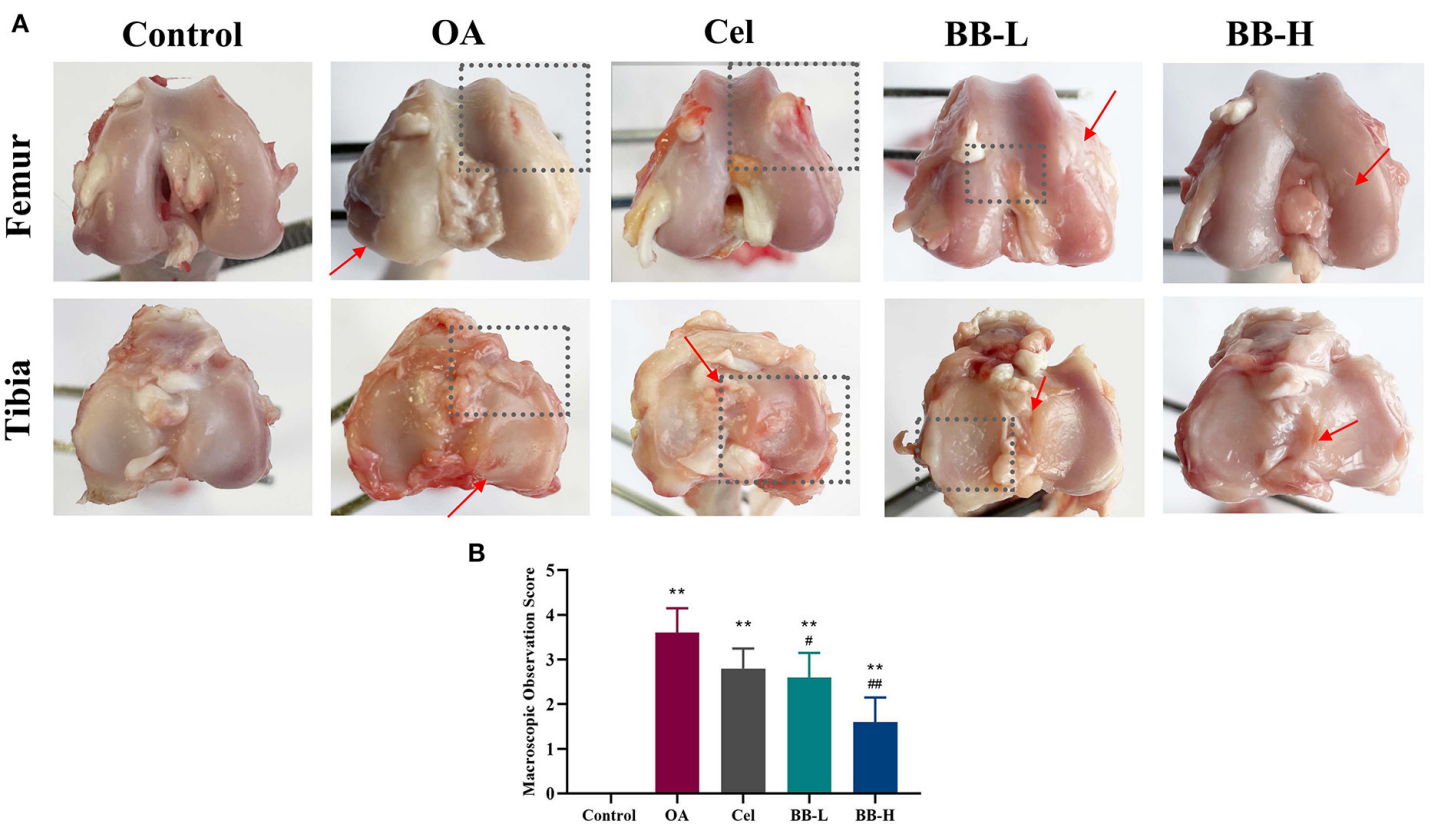
Following 8 weeks of bilobalide treatment, macroscopic images and scores for the knee joints of rabbits. **(A)** Images of the tibial plateau and femoral condyle of rabbit knee joints in each group. Arrows represent cartilage ulcers or depressions, and rectangular boxes represent areas of severe cartilage damage. **(B)** Macroscopic observation scores for knee joints in each group. All data are presented as mean ± SD (*n* = 3). ** *p* < 0.01 vs. control group; ^#^ *p* < 0.05 and ^##^ *p* < 0.01 vs. OA group.

After administering bilobalide treatment for 8 weeks, the tibial plateaus and femoral condyles of rabbits were observed macroscopically as showed in Figure 4A. In the control group, the cartilage surface was smooth and the edges were smooth and shiny. The degree of ulceration on the articular surface of the rabbits in the OA group was aggravated; the cartilage surface was damaged (rectangular box); the surface was dark red and tarnished; cartilage was eroded (arrow); the tibial edge was severely worn out, and the subchondral bone was exposed (rectangular box). The cartilage surface of the Cel group was slightly rough and thin. The surface of the femoral condyle was partially sunken (rectangular box), and the tibial surface was ulcerated (arrow). The cartilage surface of the BB-L group was rough and dull; the cartilage layer was thinned, and the cartilage surfaces of the femur and tibia were slightly rough (rectangular box). In the BB-H group, the articular cartilage was intact, and the cartilage surfaces of the femur and cartilage were slightly rough (arrows), but the tibial cartilage surface regained its luster, and the cartilage was intact on the whole. The score of macroscopic observation for cartilage was significantly higher in the OA group as compared to the control group (*p* < 0.0001). The macroscopic scores of cartilage in the BB-L and BB-H groups were significantly lower than that in the OA group (OA vs. BB-L *p* = 0.0226; OA vs. BB-H *p* < 0.0001) (Figure 4B).

### Bilobalide improves histopathological changes in the knee

**Figure 5:**
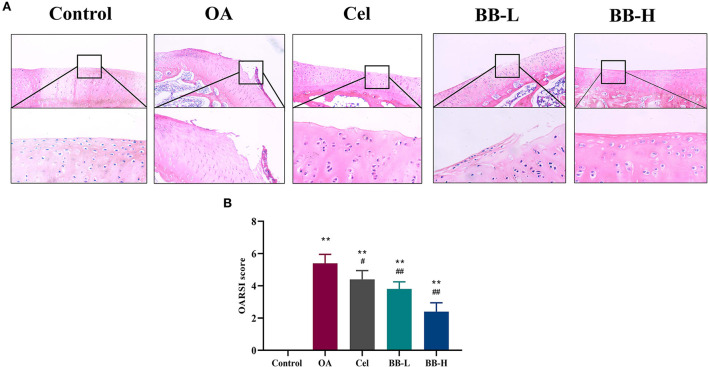
H&E staining and OARSI scores evaluation of articular cartilage for the knee in different groups. **(A)** H&E staining shows articular cartilage of the knee in different groups (200 × ). **(B)** OARSI score evaluation for different groups. All data are presented as mean ± SD (*n* = 3). ** *p* < 0.01 vs. control group; ^#^ *p* < 0.05 and ^##^ *p* < 0.01 vs. OA.

Figure 5 shows the HE-stained images (Figure 5A) and OARSI scores (Figure 5B) of rabbit knee tibias for each group. In the control group, the superficial, transition, radial, and calcified layers were placed in a columnar pattern from top to bottom correspondingly, and the chondrocytes were arranged in an orderly manner. The cartilage was significantly harmed; the articular cartilage's surface was rough; the matrix's staining intensity was diminished; apparent fissures were visible, there were fewer chondrocytes, and these were disorganized and aggregated in the OA group. The cartilage surface was rough; the surface chondrocytes were disorganized, and a few inflammatory cells had entered the cartilage layer in the Cel group. The cartilage surface of the BB-L group was rough, with an irregular arrangement of surface chondrocytes, along with hypertrophy vacuolization. In the BB-H group, the cartilage's form improved gradually; the surface chondrocytes were arranged in an orderly fashion; the surface was slightly rough but the cartilage as a whole was intact. Compared to the OA group, the Cel, BB-L and BB-H groups had significantly lower OARSI scores (OA vs. Cel *p* = 0.0226; OA vs. BB-L *p* = 0.0002; OA vs. BB-H *p* < 0.0001).

### Bilobalide up-regulates the Nrf2/HO-1 pathway in cartilage and inhibits cartilage degeneration in rabbits

**Figure 6:**
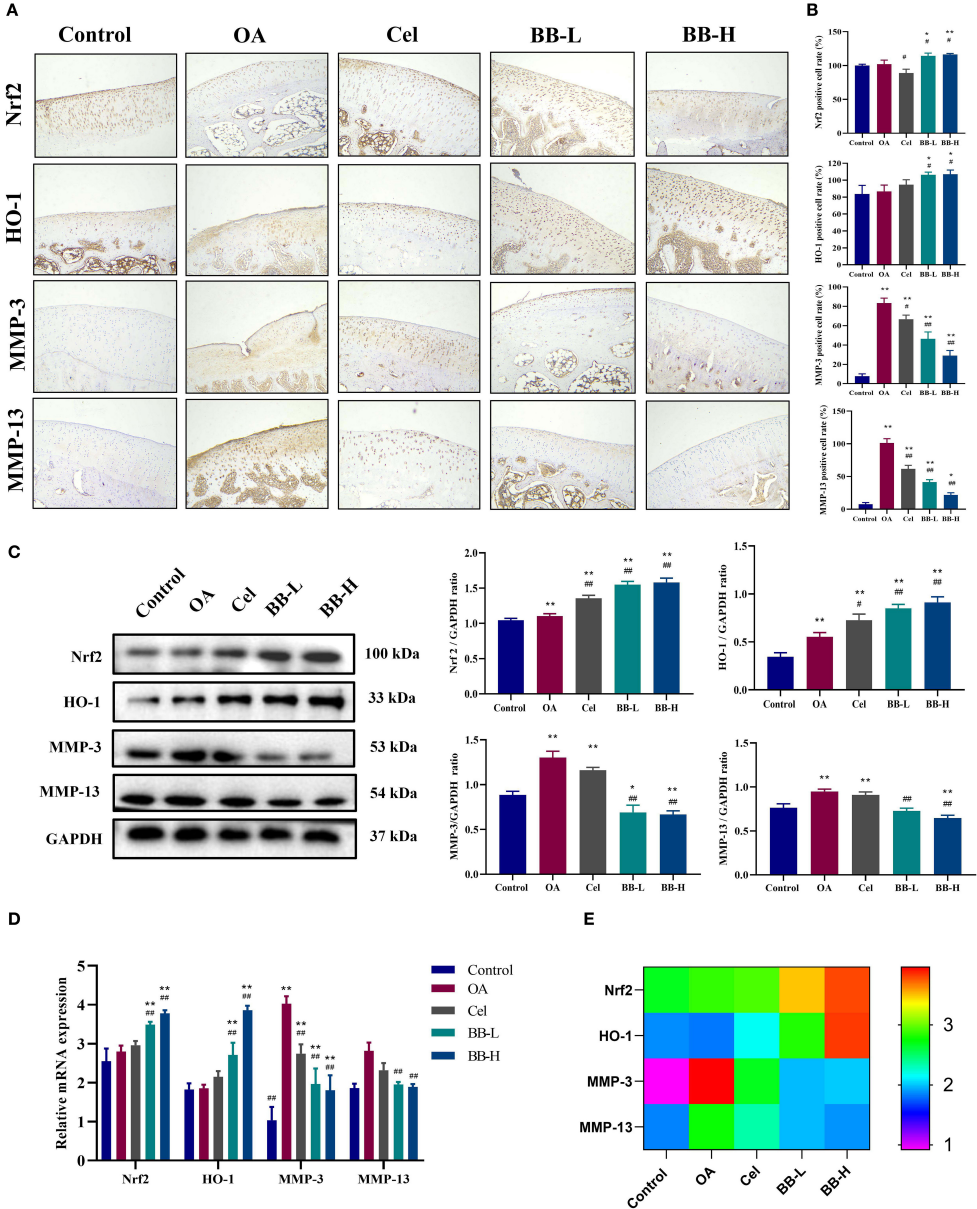
Bilobalide inhibits the production of matrix degrading proteins MMP-3 and MMP-13, *via* Nrf2/HO-1. **(A)** Representative images of immunohistochemical staining for Nrf2, HO-1, MMP-3 (200 × ), and MMP-13 proteins in rabbit cartilage in each group. **(B)** Percentage of positive cells after eight weeks of treatment. **(C)** Assessment and quantification of Nrf2, HO-1, MMP-3, and MMP-13 protein levels in cartilage by western blotting. **(D,E)** The expression of Nrf2, HO-1, MMP-3, and MMP-13 mRNA in rabbit cartilage tissue by qRT-PCR. All data are presented as mean ± SD (*n* = 3). * *p* < 0.05 and ** *p* < 0.01 vs. control group; ^#^ *p* < 0.05 and ^##^ *p* < 0.01 vs. OA group.

Figures 6A,B shows the images of immunohistochemical staining and the percentage of positively stained cells for Nrf2, HO-1, MMP-3, and MMP-13 in rabbit knee joint tissues of each group. The control group showed fewer positively-stained cells for MMP-3 and MMP-13, while the expressions of Nrf2 and HO-1 were higher in articular cartilage. As compared to the control group, the proportion of cells stained positively for MMP-3 and MMP-13 in the OA group was significantly high (*p* < 0.0001). The proportion of cells in the BB-L and BB-H groups stained positively for MMP-3 (*p* < 0.0001) and MMP-13 (*p* < 0.0001) was significantly lower than those in the OA group. Compared with the OA group, the protein expressions of Nrf2 (*p* = 0.0326) and OH-1 (*p* = 0.0335) in the BB-L group were significantly increased, and the protein expressions of Nrf2 (*p* = 0.0134) and OH-1 (*p* = 0.0278) in the BB-H group were also significantly increased. Protein and mRNA levels of Nrf2, HO-1, MMP-3, and MMP-13 in rabbit cartilage were assessed by western blotting (Figure 6C) and qRT-PCR (Figures 6D,E), respectively. Compared with the OA group, the expressions of Nrf2 (*p* < 0.0001) and HO-1 (OA vs. BB-L *p* = 0.002; OA vs. BB-M *p* < 0.0001) in the BB-L group and the BB-M group were significantly increased, while the protein expressions of MMP-3 (*p* < 0.0001) and MMP-13 (OA vs. BB-L *p* = 0.0001; OA vs. BB-H *p* < 0.0001) were significantly decreased. Similarly, the mRNA expressions of Nrf2 (OA vs. BB-L *p* = 0.0020; OA vs. BB-H *p* < 0.0001) and HO-1 (OA vs. BB-L *p* = 0.0020; OA vs. BB-M *p* < 0.0001) were significantly increased in the BB-L and BB-H groups, while the mRNA expressions of MMP-3 (*p* < 0.0001) and MMP-13 (OA vs. BB-L *p* = 0.0002; OA vs. BB-H *p* < 0.0001) were significantly decreased, which was consistent with the immunohistochemical results. Thus, bilobalide could prevent the breakdown of cartilage by reducing the levels of MMP-3 and MMP-13 by activating the Nrf2/HO-1 pathway.

### Bilobalide inhibits the expression of cartilage biomarkers and inflammatory factors in the sera of OA rabbits

**Figure 7:**
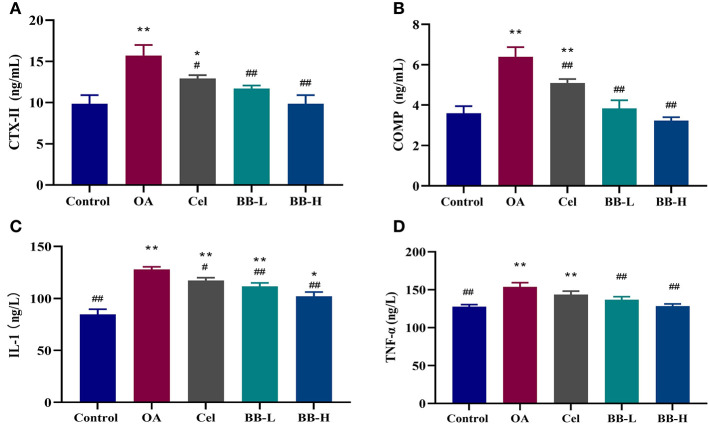
Following the administration of bilobalide for 8 weeks, the sera of rabbits in each group were tested by ELISA to measure the expressions of **(A)** CTX-II, **(B)** COMP, **(C)** IL-1, and **(D)** TNF-α. All data are presented as mean ± SD (*n* = 3). * *p* < 0.05 and ** *p* < 0.01 vs. control group; ^#^ *p* < 0.05 and ^##^ *p* < 0.01 vs. OA group.

According to the results of ELISA (Figure 7), the levels of CTX-II (*p* < 0.0001), COMP(*p* < 0.0001), IL-1 (*p* < 0.0001), and TNF-α (*p* = 0.0001) in the sera from rabbits in the OA group were significantly higher than those in the control group (*P* < 0.05), whereas the levels of BB-L (CTX-II: *p* = 0.0021; COMP: *p* < 0.0001; IL-1: *p* = 0.0018; TNF-α: *p* = 0.0033) and BB-H (CTX-II: *p* < 0.0001; COMP: *p* < 0.0001; IL-1: *p* < 0.0001; TNF-α: *p* = 0.0001) in the Cel group (CTX-II: *p* = 0.0233; COMP: *p* = 0.0060; IL-1: *p* = 0.0316; TNF-α: ns), were significantly lower than those in the OA group, demonstrating that bilobalide could reverse OA-induced elevation of CTX-II, COMP, IL-1, and TNF-α levels.

## Discussion

OA is caused by an imbalance in the synthesis and breakdown of articular cartilage and is a key factor for disability (30). At present, its pathogenesis is unclear but, involves changes in the microenvironment of the entire joint tissue (31). Natural products with cartilage repair, anti-inflammatory, and antioxidant properties are also widely used to prevent or treat OA (32, 33). In this study, we found that bilobalide inhibited the protein levels of papain-induced MMP-3 and MMP-13 in rabbit cartilage tissue and the expressions of IL-1, TNF-α, CTX-II and COMP in sera by up-regulating the Nrf2/HO-1 pathway in the cartilage. Bilobalide also inhibited the destruction of the 3D structure of the trabecular bone and alleviated bone erosion, thereby preventing the pathological processes of OA in rabbits.

Animal models of OA are used to precisely duplicate the extent and progression of joint destruction, thus allowing researchers to pinpoint the underlying causes of symptoms, and the course of the illness, and explore potential therapeutic targets (34, 35). The rabbit OA model has been widely used to assess the efficacy of various compounds, and it allows the study of the underlying mechanisms in ways not possible in humans (36, 37). In order to observe the effect of bilobalide on early OA, we selected 3-month-old rabbits whose cartilage was in the growth phase. In the papain-induced OA model, for more than 2 weeks, the articular cartilage suffered minor damages only but its pathology changed considerably, and the low level of inflammation was consistent with the early pathological changes. Therefore, we administered bilobalide for 2 weeks after modeling. The protective effects on early OA were evaluated by observing the rabbits gavaged with bilobalide for 8 weeks. The rabbit OA model used in this study is a good translational model for the initial screening of compounds to evaluate disease mechanisms or treatments. Bilobalide could suppress pathological damage and degeneration of cartilage and markedly reduce the expression of the matrix degradation proteins, MMP-3 and MMP-13, suggestive of its protective effects against cartilage destruction. Notably, we assessed the effects of bilobalide on bone remodeling in a rabbit model of OA to assess its preventive activity on various physiological features of OA.

An imbalance between cartilage degradation and repair mechanisms is the primary cause of the early-onset OA (35, 38). However, some studies indicate that synovitis and subchondral bone remodeling may occur before the joint deterioration in the early phases of OA (39). Due to enhanced bone remodeling, OA is linked to early bone loss, sluggish turnover, densification of the subchondral plate, and total cartilage loss (40). Osteoclast bone resorption and osteoblastic bone production are coupled during bone remodeling, replacing the damaged bone with a new bone (41). As compared to the control group, the percentage bone volume (BV/TV), mean trabecular number (Tb.N) and mean trabecular thickness (Tb.Th) of the OA group decreased significantly, and the mean trabecular separation (Tb.Sp) increased. This may be because bone catabolism is greater than its anabolism in the early stage of OA, and the subchondral bone mass decreases. The increase in Tb.Sp parameter indicated an increase in the average width of the medullary cavity between trabecular bone, suggesting that bone resorption was activated in the subchondral bone microenvironment. After bilobalide intervention, as compared to the OA group, BV/TV, Tb.N and Tb.Th increased significantly, while Tb.Sp decreased substantially, indicating that bilobalide could delay the process of bone resorption and inhibit the destruction of the 3D structure of trabecular bone.

Increased oxidative stress and age-related changes in mitochondrial antioxidant capacity may cause abnormalities in physiological cell signaling that jeopardize cellular integrity and accelerate aging (42, 43). The function of Nrf2 transcription factors in cartilage homeostasis and aging has attracted more attention recently (14, 44). A significant downstream target of Nrf2, HO-1 is essential for maintaining the redox balance in cartilage (45). *Ginkgo biloba* L. extract exerts antioxidant effects against several disorders (46–48). Western blotting showed that the expressions of HO-1 and Nrf2 increased markedly in the papain-induced OA model, indicating that the Nrf2/HO-1 pathway was affected by oxidative stress. It was activated but insufficiently resistant to the occurrence of cartilage damage. Different doses of bilobalide could not only alleviate the pathological changes in the OA model but the expressions of Nrf2 and HO-1 in the BB-L and BB-H groups increased further and showed better performance than in the Cel group. MMP-13 and MMP-3 are major proteases involved in the degradation of the extracellular matrix of cartilage (49). It has the special ability to cleave type II collagen or non-collagen (50, 51). As compared to the OA group, MMP-3 and MMP-13 decreased significantly after gavage for 8 weeks. We hypothesized that bilobalide's specific modulation of the Nrf2/HO-1 pathway was responsible for the decline in the levels of matrix degradation protein. This implied that bilobalide prevented matrix breakdown by stimulating the Nrf2/HO-1 axis, which in turn suppressed oxidative stress in the cartilage.

OA biomarkers have been widely used to diagnose OA, measure the extent of pathological progression, and assess the effectiveness of drugs (52, 53). Studies have shown that the type II collagen metabolites, CTX-II, and the non-collagen metabolite, COMP, in the extracellular matrix can be used to assess the extent of cartilage damage (54). The levels of CTX-II and COMP in the OA group increased significantly after modeling as compared to the control group, indicating that during cartilage degeneration, the breakdown of extracellular matrix was accelerated and CTX-II and COMP entered the blood, resulting in disorders of cartilage metabolism. Previous studies have shown that treatment with 10 mg/kg glycyrrhizin significantly inhibited the levels of CTX-II in OA rats (55). Rats without ovaries expressed less CTX-II and COMP in their sera for 9 weeks when administered 51 mg/kg of soybean isoflavones (56). Treatment for 7 days with 4 mg/kg undenatured type II collagen in an intragastrically administered of iodoacetic acid-induced rat OA model, resulted in an effective reduction in the expression of COMP in the serum (1). After administration of bilobalide, the levels of CTX-II, COMP, IL-1, and TNF-α in rabbit sera reduced significantly, indicating that bilobalide could inhibit the degradation of extracellular matrix and exert chondroprotection through anti-inflammatory and anti-extracellular matrix degradation effects.

Bilobalide exerted antioxidant and anti-matrix degradation effects by activating the Nrf2/HO-1 pathway in the cartilage, thereby inhibiting and improving cartilage degeneration in rabbits. It could also repair papain-induced cartilage loss and subchondral bone injury in rabbits with OA. In the early stages of OA, bilobalide improved the biomechanical characteristics and microstructural alterations of the subchondral bone. However, only *in vivo* tests were conducted in this study to assess the anti-OA impact of bilobalide. Further, *in vitro* research is therefore required to clarify the molecular mechanism underlying bilobalide's effects on subchondral bone remodeling. Rigorous toxicity tests and pharmacological experiments are, required to ascertain the long-term effectiveness of bilobalide in the treatment of OA. Nevertheless, the protective mechanism of bilobalide on articular cartilage may be better understood as a result of our observations.

## Data availability statement

The original contributions presented in the study are included in the article/supplementary material, further inquiries can be directed to the corresponding author/s.

## Ethics statement

The animal study was reviewed and approved by Laboratory Animal Welfare and Ethics Committee of Northeast Agricultural University (#NEAU-2022-01-0025-1).

## Author contributions

TM, HC, and LG conceived and designed the experiments. HR, LL, JZhao, YY, and LJ performed the experiments. TM, HR, XL, YZ, and XX analyzed the data and prepared figures. TM, HC, and JZhang wrote the manuscript. All the authors read and approved the final manuscript.

## Funding

This work was supported by Inner Mongolia Autonomous Region Science and Technology Project (2022YFSH0052), Xinjiang Uygur Autonomous Region Science and Technology Support Project Plan (2020E0236), Applied Technology Research and Development Plan of Heilongjiang (GA18B203), and National Science and Technology Major Projects and Key R&D Projects Provincial-level Funded Projects (GX18B023).

## Conflict of interest

The authors declare that the research was conducted in the absence of any commercial or financial relationships that could be construed as a potential conflict of interest.

## Publisher's note

All claims expressed in this article are solely those of the authors and do not necessarily represent those of their affiliated organizations, or those of the publisher, the editors and the reviewers. Any product that may be evaluated in this article, or claim that may be made by its manufacturer, is not guaranteed or endorsed by the publisher.
